# Promoting Diversity in the Clinical and Translational Research Workforce

**DOI:** 10.1016/j.jnma.2018.03.010

**Published:** 2018-05-10

**Authors:** Estela S. Estape, Alexander Quarshie, Barbara Segarra, María San Martin, Ruth Ríos, Karen Martínez, Jacquelyn Ali, Ulochi Nwagwu, Elizabeth Ofili, Priscilla Pemu

**Affiliations:** Estela S. Estape, School of Health Professions, Medical Sciences Campus, University of Puerto Rico (MSC-UPR), United States; Alexander Quarshie, Morehouse School of Medicine (MSM), United States; Barbara Segarra, School of Health Professions, Medical Sciences Campus, University of Puerto Rico (MSC-UPR), United States; María San Martin, School of Health Professions, Medical Sciences Campus, University of Puerto Rico (MSC-UPR), United States; Ruth Ríos, Graduate School of Public Health, Medical Sciences Campus, University of Puerto Rico (MSC-UPR), United States; Karen Martínez, School of Medicine (SoM), Medical Sciences Campus, University of Puerto Rico (MSC-UPR), United States; Jacquelyn Ali, Morehouse School of Medicine (MSM), United States; Ulochi Nwagwu, Morehouse School of Medicine (MSM), United States; Elizabeth Ofili, Morehouse School of Medicine (MSM), United States; Priscilla Pemu, Morehouse School of Medicine (MSM), United States

**Keywords:** Diversity, Research workforce, Clinical and translational research, Health disparities, Minority health

## Abstract

The positive impact of diversity in increasing the effectiveness of the research workforce has been undeniably demonstrated to be an essential element for achieving health equity. Diversity is also instrumental for the research workforce to advance discovery, eliminate health disparities, improve minority health and achieve effective patient-centered outcomes in the quest for better health. One of the sustainable ways to achieve diversity in the work force is through training, education and career development of all interested individuals including minority, underserved, underrepresented and populations with special needs. A Hispanic public, academic health center, and a historically black private medical school, have joined efforts in this article to share their experiences in addressing diversity in the clinical and translational research workforce with grant support from the National Institutes of Health. The purpose of this paper is to describe how diversity has been achieved through a concerted effort to recruit and develop underrepresented junior faculty and doctoral candidates for successful careers in clinical and translational research focused on health disparities and minority health. We describe Initiatives designed to achieve diversity in recruitment and development of research teams, together with an evaluation of outcomes to determine the success of the program and its participants.

## INTRODUCTION

There is a critical need to increase and diversify the biomedical research workforce, as achieving diversity is crucial to the full accomplishment of the national research goals to improve health, access, and quality of care.^1^ To help attain this purpose, the National Institutes of Health (NIH) is the primary funder of research training and career development for clinicians and physicians in the United States of America (USA).^2^ As such, numerous and diverse training and career development awards have been developed by NIH to empower the next generation of clinical and translational researchers, including minority, underserved and underrepresented populations.^3^ The NIH created the Clinical Research Education and Career Development (CRECD) R25 award in 2001, to increase the participation of under-represented scientists in the nation’s biomedical research enterprise. The CRECD award includes support for minority-serving institutions to develop and offer a formal 2-year research degree program (Phase I). It also supports additional research mentored training experiences of 1–2 years (Phase II) to a limited number of outstanding candidates from minority and underrepresented groups that completed Phase I successfully.

The University of Puerto Rico- Medical Sciences Campus (UPR-MSC), a Hispanic public, academic health center, and Morehouse School of Medicine (MSM), a historically black private medical school, were the first CRECD funded programs in the nation. Both institutions have in common several key factors that facilitate diversity both in their recruitment and scientific enterprise: an academic environment where the majority of the leaders and faculty are from underrepresented minority groups in the USA; strong partnerships and collaborations with other institutions and specific criteria to measure program and Scholars’ outcomes.^4–6^ The long-term goal of both programs is to increase the diversity in gender, race/ethnicity, and disciplines participating as clinical and translational researchers, with the skills and competencies to actively lead successful multidisciplinary research teams. This new set of translational research competencies have been developed in response to the identified need to facilitate the transfer of knowledge into action.^7,8^ Collaboration between disciplines, researchers and end users of research, such as patients and healthcare stakeholders, can increase the capacity to address the research-practice translational gap.^9^

## METHODS

### Institutional background

The Phase I of the NIH-CRECD award at the UPR-MSC includes completing a post-doctoral Master of Science in Clinical and Translational Research (MSc) program. This graduate program was developed in collaboration with Mayo Clinic School of Medicine, Rochester, MN.^4^ The MSc was the first joint degree in the UPR-MSC offered between two schools: Health Professions and Medicine. Having shared leadership between the two schools has been an effective strategy to increase knowledge about other health professions, research interests and enhance collaboration among different disciplines. Consonant with this goal, the MSc pool of candidates and their professional diversity expanded with each grant cycle. At the beginning of the program, recruitment efforts targeted potential candidates (physicians, clinicians, and health-related degrees) from the six Schools at the Medical Sciences Campus, UPR, including the international exchange program of the UPR-MSC School of Dental Medicine. The scope of the program’s recruitment was increased with each cycle of funding to reach interested candidates with doctoral degrees from other UPR Campuses, as well as from other accredited academic institutions of higher education in Puerto Rico. The MSc program also accepted applications from candidates of the Puerto Rico Department of Health and the San Juan Veterans Administration.

Morehouse School of Medicine (MSM) is a historically black institution ranked number one in social mission among all USA medical schools,^10^ with an established record of recruiting and nurturing underrepresented minority students in its training programs. MSM CRECD Phase I award includes the Master of Science in Clinical Research (MSCR) degree program established as a broad-based multi-disciplinary graduate-level program in clinical and translational research (CTR). The program was designed to prepare doctoral and post-doctoral scholars for a career in clinical and translational research. The CRECD MSCR program at MSM aligns with the national commitment to train the next generation of clinical and translational scientists by recruiting pre-doctoral and post-doctoral scholars. The MSCR program collaborates with the Atlanta Clinical and Translational Science Institute (ACTSI) which is a Clinical and Translational Science Award (CTSA) program at Emory University, Morehouse School of Medicine and Georgia Institute of Technology.^11^ During its first five years, the MSCR program focused on developing a critical mass of CTR scientists, by recruiting and retaining talented junior faculty, who are likely to advance independent careers in biomedical research. During the subsequent five years, the program was strategically expanded to include MD and Ph.D. pre-doctoral trainees, as well as post-baccalaureate Masters level candidates. This strategy was designed to increase the pipeline of CTR researchers to enhance the diversity of the work-force, and to build multidisciplinary teams. The partnership with the Atlanta Clinical and Translational Science Institute (ACTSI) has supported this strategic program expansion through additional funding using the NIH TL1 (MD and Ph.D. pre-doctoral trainees) and KL2 (MSM junior faculty scholars) mechanisms, by increasing access to mentors and sharing of coursework.

The philosophy guiding the efforts of both programs has remained constant: to develop multidisciplinary scientific teams working in collaboration for the translation of research findings to improve quality of life. We believe that the design of these programs will help to address the critical need to replenish and diversify the biomedical research workforce through a concerted effort to recruit and train junior faculty clinicians and scientists, pre-doctoral students, post-doctoral students and post-baccalaureates for successful careers in clinical and translational research.

### Recruitment strategies

There has been a positive impact of the NIH CRECD program in developing initiatives in our respective institutions by facilitating recruitment, retention, and to achieve diversity in the candidate’s pool with benchmarks defined for measurement of success. The dissemination and recruitment efforts are a dynamic process that has evolved with time, fulfilling the needs for an increase in the use of technology for effective communication and are not unique to our programs. Orientation activities are simultaneously transmitted through the modality of Go-To-Meeting to facilitate remote participation. The program’s information is disseminated through the program’s website and brochure, promotional flyers, posters, electronic communications and presentations at student orientation activities. Also, the CRECD program hosts seminars and supports faculty participation in research career development events, to increase awareness of the program.

Other outreach efforts include targeted e-mails, presentation of workshops at departmental noon conferences and word of mouth from our students and graduates. Both Institutions encourage clinical and basic science departmental chairs, as well as directors of the various training programs, to identify prospective candidates. Also, we pursue new innovative collaborative recruitment strategies such as expanding our pool of mentors through our respective collaborators and partners. For example, MSM also addresses diversity by targeting Historically Black Colleges and Universities (HBCUs) and diverse student organizations at predominantly white institutions. Staff associated with the MSCR program host presentations at school campuses in different states, attend national recruitment events, in particular, activities garnered for HBCUs, and conduct outreach to minority sorority and fraternity organizations. The MSc has established partnerships across the island with two UPR campuses and four private institutions that offer doctoral degrees to expand recruitment, and their candidates compete for CRECD Scholars’ support.

In addition to recruitment activities, we implemented strategies to increase the interest in the programs of outstanding minority candidates from diverse disciplines through different approaches including:

- Commitment and enthusiasm demonstrated by successful program graduates in becoming mentors, co-mentors and role models for the incoming scholars.
- Increasing the number of hybrid and online courses^12^
- Recruitment of teaching faculty from diverse disciplines.
- Providing scholars incentives and awards to cover program’s registration fees, research expenses, travel, career development opportunities and salary support for release time

To enhance public understanding and to increase other students and faculty’s interest in research and workforce development, the CRECD Programs support the participation of faculty, scholars, and graduates in scientific meetings, outreach and networking efforts and other endeavors that help us disseminate outcomes and achievements. For example, Friday and Thursday Seminars have been developed in the MSc and MSCR respectively, as venues to disseminate the programs’ accomplishments and opportunities for career development. These seminars expose the scholars to successful clinical researchers and experts in the field of clinical research and health disparities and allow the discussion of topics relevant to their development as clinical and translational researchers.

Student retention is critical to the program’s success. Also, mentoring and guided participatory learning are the most successful approaches for biomedical research faculty development programs.^13^ The following strategies are used to ensure scholar engagement and retention:

- Mentors are required to establish regular contact with scholars through a monthly meeting to review their status and progress toward fulfilling the scholars’ goals.
- Mentors prepare semi-annual written evaluations utilizing standard forms created for this purpose. Scholars, on the other hand, submit progress reports. These are shared either with the scholar or mentor respectively and with the program directors.
- Scholars and Lead Mentors meet with program directors every six months (and as needed) to review progress, identify problems that may be interfering with their development, and determine potential solutions.

Upon completion of the MSCR program, each scholar prepares a summary of his or her research experience, including accomplishments, plans, and a critique of the program. The MSc program performs an Annual Evaluation Retreat with the scholars to gather their experiences and recommendations through the use of questionnaires, presentations and discussion. These data are reviewed, presented to internal and external advisory committees of the program, and programmatic changes are made as applicable.

## RESULTS AND DISCUSSION

### Benchmarks for program’s success

The strategies previously described to increase diversity in the applicant’s pool are consistent with the following program’s evaluation benchmarks for success:

1. Multidisciplinary career development:
  - maintaining diversity in the Scholars’ disciplines admitted in each cohort
  - requiring diverse disciplines in the Scholars’ research committee composition
  - promoting research in different areas of health disparities.
2. Multi-institutional participation:
  - sponsoring enrollment of scholars from more than one partner school,
  - supporting the involvement of school partners in the programs’ Advisory Committee
  - inviting partner school’s faculty to participate in the programs’ Admissions Committee.

**Figure 1:**
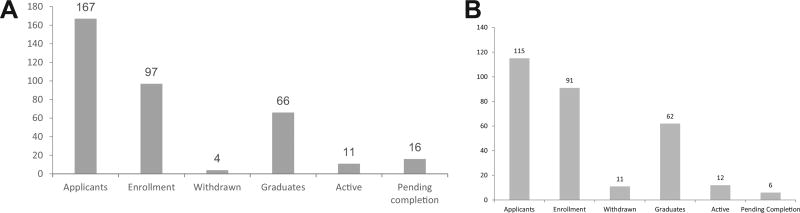
A. Distribution of applicants and matriculants of UPR MSc program. B. Distribution of applicants and matriculants of MSM MSCR program.

One of the direct benchmarks used to evaluate the achievement of the program’s diversity outcomes is through the analysis of the annual enrollment of Scholars. The CRECD award provides funding to support a maximum of 3–4 Scholars per year to participate in Phase I (approximately 60 scholars in a period of 15 years). Nevertheless; each program has identified other alternatives of financial support to offer the opportunity to competent and qualified candidates. This additional support is one of the reasons for the observed increase in the enrollment of both programs: MSCR (91) and MSc (97). Figure 1A and B presents the number of applications received, enrollment, withdrawals, graduates, active and pending completion for each program during the first 15 years of funding.

The MSc and MSCR programs have a duration of 2–3 years with similar requisites for graduation: the completion of a research project and submission of a first author manuscript. Both programs had retention rates over 85% (88% MSCR and 96% MSc) and Scholars who withdrew from the programs within the first months of admission were due to personal reasons. Also, both programs have a similar number of graduates and active scholars (within the 2- year curriculum). The MSc has sixteen scholars pending to complete the requirement of manuscript submission, resulting in a completion rate for UPR (71%) when compared to MSCR (78%) with six pending completion.

**Figure 2:**
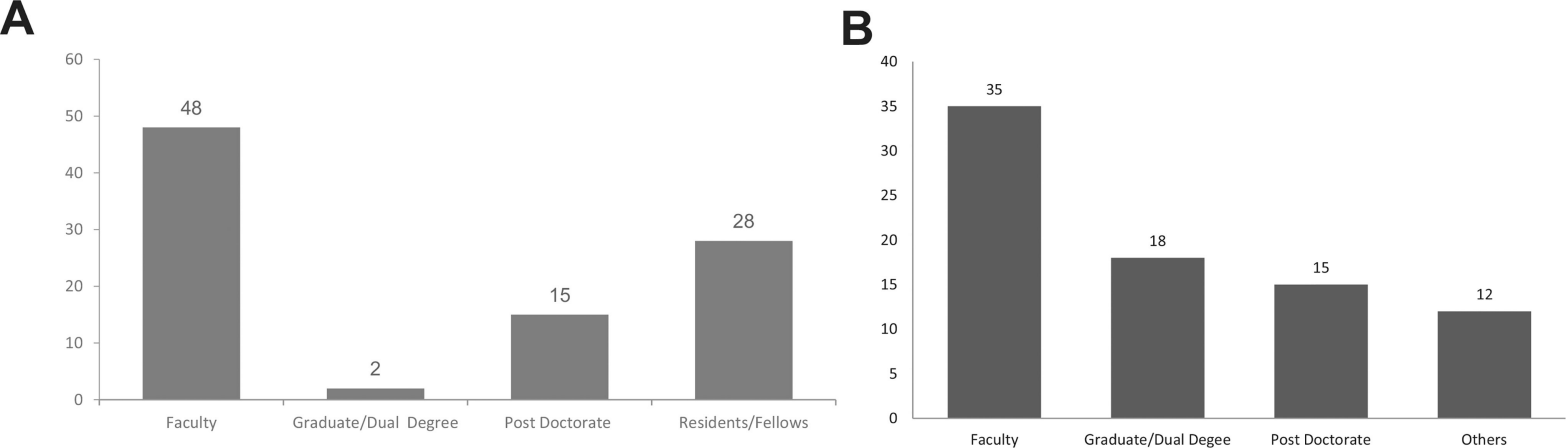
A. Career stage of the UPR MSc applicants at admission. B. Career stage of the MSM MSCR applicants at admission.

Figure 2A and B show the career stage of the applicants at the time of admission to each program. In both programs, faculty scholars had the highest percentage in enrollment (MSCR 44% and MSc 51%). The MSc program maintained its focus on the recruitment of postdoctoral candidates from diverse disciplines including MD, DMD and PhD, with 30% of residents/fellows and 16% doctorate not occupying faculty positions. MSCR expanded to include MD and PhD pre-doctoral trainees (graduate/dual degree), as well as post-baccalaureate Masters level candidates (others).

The profile of the scholars admitted to the MSc (n = 93) and the MSCR (n = 80) is presented in Tables 1A and B. Both programs were successful in the inclusion of women in research as well as in the enrolment of health professionals with diverse disciplines and backgrounds. As shown, more than 50% of women were admitted (MSc 62% and MSCR 77%) and every cohort included different disciplines with the MDs and PhDs being the largest two groups represented. All Scholars belong to minority and underrepresented populations in science: Hispanic or African American. Also, all the Scholars’ research projects addressed health disparity priorities, and their research committees included mentors from at least two disciplines. The programs have identified health disparities areas that the Scholars are required to select from, and develop their research project. Nonetheless, many of the projects include more than one area of health disparities in one single project: examples include oral health and cardiovascular; aging and mental health; cancer and obesity.

### Benchmarks for graduates’ success

The programs’ benchmarks for success go hand in hand with the CRECD graduates’ benchmarks for success in their research career development: scientific presentations; peer-reviewed and public domain publications; honors and awards; submission of grants and externally funded research projects; research -related and/or academic appointments.

**Figure 3:**
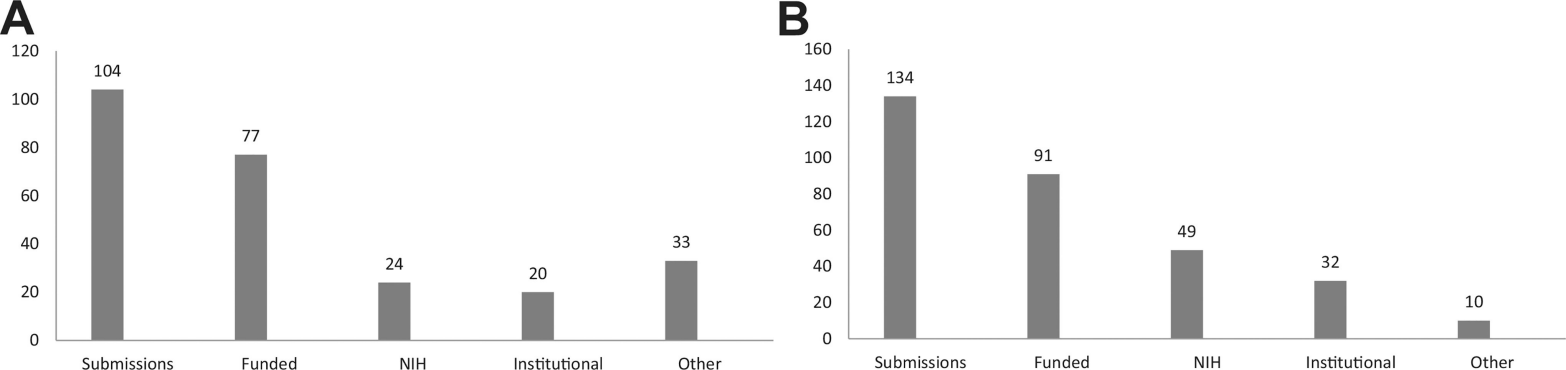
A Grant productivity of UPR MSc Scholars Cohorts 2003–2012. B. Grant productivity of MSM MSCR Scholars Cohorts 2002–2011.

Figure 3A and B present a 10-year Cohort summary of the respective CRECD Scholars’ outcomes regarding grant productivity: UPR-MSc program (2003–2012) and MSM-MSCR program (2002–2011). Presenting the grant productivity data for the first 10 years of the programs allows the inclusion of scholars with at least three years after graduation. This timeframe gives the scholars opportunity to publish and submit grants, as well as provides the programs a reasonable time to evaluate their grant productivity, including total of grants submitted, funded and the funding source (NIH, institutional, government, foundations and other support, such as industry).

Of a total of 104 grants submitted by the MSc graduates, 77 (74%) were funded for a total of approximately 19.1 million. Of these, 24 (23%) were funded by NIH. The type of NIH grants funded were: 13 (54%) R series research grants, including 3 RO1s, 2 R21, 4 pilot projects, 1 R25 and 1 Supplement. There were 5 research training and career development awards (K99/R00, K12, T32, G12 and an LRP) and 6 program projects and center grants. Institutional grants are from internal sources such as Departments, Schools and Endowments. Other include external resources from the local government, such as the Department of Health; and national level including funding from DoD, SAMSHA and CDC.

The MSCR graduates that enrolled as junior faculty over the first ten years of the program have made a total of 134 grants submissions, and received funding for 91 (68%) for a total of approximately 43 million. Out of the 91 grants funded, 56 (61%) were pilot grants, and 49 (54%) were federal/government funded.

The funding rate gives a percent of the number of individual investigators who are seeking and getting funding. In NIH, it is calculated by dividing the number of PIs funded by the number of PIs who apply in any given fiscal year. The funding rate for first-time RO1 investigators that applied to NIH and were successful in 2016 was 20%, that is only 1 out of 5 applications submitted was funded.^14^ Therefore, obtaining grant funding is a challenge that requires individual persistence, institutional support and writing skills for publication and grant re-submission. Both MSCR and MSC programs’ leadership are aware of the competitive environment faced by the Scholars after graduation and recognize the importance of the CRECD Phase II to support those graduates that submit grants and are committed to become independent investigators.

### Building the next generation of minority research leaders

The current description of the National Institute on Minority Health and Health Disparities (NIMHD) CRECD program awards clearly states their goal to “support creative and innovative research education programs that promote the development of well-trained clinical researchers who can lead clinical and translational research”.^15^ Consonant with this goal, our Masters programs continue to enhance the curriculum with the latest scientific advances and approaches to expand institutional and national research capacity and infrastructure to address minority health and reduce health disparities. We are building the next generation of minority research leaders that will join the workforce in different capacities with the skills and knowledge to lead multidisciplinary teams. Through December, 2017, our Masters programs admitted a total of 191 Scholars, of which 142 (74%) are women, a significant addition of women scientists to the current research workforce in which the number of women is significantly under represented. We are including a brief description of the career pathway of two graduates from our programs who exemplify the above: they are both from an underrepresented population, women, physicians, active researchers and have currently joined our programs in leadership positions.

Karen G. Martínez, is a practicing child psychiatrist and Assistant Professor in the UPR Department of Psychiatry, School of Medicine. Dr. Martínez was a CRECD MSc Phase I Scholar from 2006 to 2008 and continued as a Phase II Scholar (2008–2010). During Phase I, she developed and published a manuscript on the role of maternal depression in pediatric asthma. As part of Phase II, Dr. Martínez transitioned into the area of research of fear and anxiety with a new mentor, publishing 4 peer-reviewed articles on translating fear extinction beyond anxiety disorders. As part of her career development, she applied and obtained support from the competitive NIH Loan Repayment Program, a Pilot Project, service proposal from Susan G Komen and a Phase III award (2013–2017). This last one is an institutional award for exceptional Phase II candidates supported by the UPR Endowment program Hispanics in Research Capability (HiREC). During this period, she developed the Center for the Study and Treatment for Fear and Anxiety at the UPR-MSC where Dr. Martínez trains undergraduate, graduate and medical students as well as psychiatry residents on clinical translational research in fear and anxiety. At this Center, she has been able to culturally adapt evidence-based treatments for anxiety, published 2 manuscripts and obtain preliminary data for an R01 proposal that was submitted but not funded. She continues to work on a revised proposal for an RO1 evaluating the role of cultural symptoms such as “ataque de nervios” on the development of Post-traumatic stress disorder (PTSD). She has received multiple awards recognizing her research work including the Career Development Award from the Anxiety Disorders of America, and a Minority Faculty Award from the American College of Neuropsychopharmacology. On July 2016, she was appointed multiple PI and Scientific Director of the UPR-MSC CRECD program. Dr. Martínez was responsible for directing the efforts of writing the application for the CRECD R25 grant renewal, which was granted for an additional 5 years (2017–2022).

Priscilla Pemu is a physician who enrolled in the CRECD MSCR program after internal medicine residency in 2002 at the rank of Assistant Professor. She completed Phase 1 in 2004 with 2 grant applications submitted (NIH K-08, American Heart Association Beginning grant in aid: none funded),4 manuscripts published, and an institutional career development award to explore the mechanistic basis for vascular dysfunction in obesity. In the next 5 years, up to 2009, she submitted 2 grant applications (CCRE subproject PI; META Health NHLBI both funded) successfully published 8 manuscripts, served as Associate Program Director for the Internal Medicine residency program and was promoted to Associate Professor. She developed her work with the Practice Based Research Network known as the Community Physicians’ Network (CPN)where she was responsible for quality improvement initiatives for hypertension, diabetes and metabolic syndrome. In the next 5-year period up to 2014, she was funded by Phase II CRECD, published 6 manuscripts, submitted 5 grant proposals with 2 funded (NIDDK R34; AHRQ R18 (Funded); PCORI disparities application; NIMHD R01 and NIMHD Transdisciplinary Collaborative Center for Health policy (funded)). She started to direct the Clinical Trials course and was promoted to full Professor. She has mentored 14 trainees at various levels (medical student/PhD/Medical residents), and currently participates as a co-investigator in the National Research Mentoring Network (NRMN) funded by NIGMS. For the next 5-year period through 2019; so far, she is site PI for 2 large multi-center NIH clinical trials (SPRINT and ASPREE), submitted 5 grant applications as part of a collaboration (AHA (funded); multiple PI of the NIH All of US Research Program (funded); NIDDK Center for Diabetes translation (funded); BIRCHW T32 (funded); and NIMHD TCC for health disparities (not funded). She serves as scientific reviewer for the American Cancer Society and American Heart Association. She is Multi PI for the CRECD program at Morehouse School of Medicine. Her trajectory is a reflection of the program at Morehouse: it provided a supportive environment with senior mentors, opportunities for collaborations and writing accountability groups led by a seasoned mentor.

## IMPLICATIONS

The NIH CRECD R25 program has provided the opportunity to build an efficient pathway to achieve a collaborative environment that can meet the needs of dynamic healthcare systems by increasing diversity. Through this program, we increased the number of total grant applications from two groups that are highly underrepresented in NIH research awards: Hispanics and Black or African Americans.^16^ A unique attribute of our CRECD programs is the establishment of research career development programs in institutions whose historical mission has been educating and training individuals from underrepresented groups in the biomedical sciences. Keeping this framework in mind, it has helped us to create, maintain, expand and strengthen our research education and career development programs to achieve the following goal:

- *To support the creation of a diverse health workforce that includes clinical-translational minority researchers with the capacity to help advance the integration of different disciplines in a team for the benefit of accelerating dissemination and implementation of new knowledge into health care services and policy to eliminate health disparities and achieve health equity*.

Our results show that post-doctoral research training programs have a positive impact on the health research workforce diversity by increasing by 134 graduates the number of clinical translational researchers from minority and underrepresented populations. Also, the diversity among the post-doctoral health professionals selected each year, promote collaboration among and across disciplines and enhances a multi-disciplinary teamwork vision towards research.

This diverse workforce is needed to efficiently address the challenges posed by the interest of the public in general and scientific advances such as those seen in health communication, technology, information systems and precision medicine. Diversity in discipline, the area of study, gender, and multidisciplinary research committee, have been critical factors for our scholars to experience and understand team science and its importance in eliminating health disparities.

### Future directions

The CRECD program will expand collaboration with national workforce programs such as the CTSAs. The opportunity to increase funding success for career development and R01 awards will leverage collaboration with the NIH Diversity Program Consortium, and specifically, the National Research Mentoring Network (NRMN), and collaboration with the National Medical Association (NMA) and the NMA Montague Cobb Institute Scholars.

## Supplementary Material

Supplemental

## Figures and Tables

**Table 1:** 

A. UPR MSc scholars 2003–2017.		
Scholars’ Characteristics (n = 93)	Percent (%)	Frequency (n)
**Gender**		
Male	37.6	(35)
Female	62.4	(58)
**Discipline**		
MD	44.1	(41)
PhD	30.1	(28)
DDS/DMD	16.1	(15)
Other	9.7	(9)
**Race/Ethnicity**		
Latino or Hispanic	100%	93
**Research Area of Interest**		
Cancer	30.1	(28)
Oral Health	17.2	(16)
Mental Health	16.1	(15)
Cardiovascular Diseases (CVD)	7.5	(7)
Aging	7.5	(7)
Diabetes	6.5	(6)
HIV	6.5	(6)
Asthma	6.5	(6)
Other Health Disparities’ Topics	2.1	(2)

B. MSM MSCR scholars 2002–2017.		
Scholars’ Characteristics (n = 80)	Percent (%)	Frequency (n)
Gender		
Male	30.0	(24)
Female	70.0	(56)
**Discipline**		
MD	37.5	(30)
PhD	5.0	(4)
DDS/DMD	2.5	(2)
Other	55.0	(44)
**Race/Ethnicity**		
Asian, non-Hispanic	15.0	(12)
Latino or Hispanic	2.5	(2)
Black or African American, non-Hispanic	78.8	(63)
White, non-Hispanic	3.8	(3)
**Research Area of Interest**		
Cancer	22.5	(18)
Cardiovascular Disease (CVD)	12.5	(10)
Other Health Disparities’ Topics	8.8	(7)
Diabetes	8.8	(7)
Infectious Diseases	6.3	(5)
Women’s Health	6.3	(5)
Pediatrics	5.0	(4)
Sickle Cell Disease	3.8	(3)
Sleep/Circadian Rhythm	5.0	(4)
Oral Health	2.5	(2)
HIV	6.3	(5)
Malaria	6.3	(5)
Smoking	6.3	(5)
